# The application of ^2^H_2_O to measure skeletal muscle protein synthesis

**DOI:** 10.1186/1743-7075-7-31

**Published:** 2010-04-21

**Authors:** Heath G Gasier, James D Fluckey, Stephen F Previs

**Affiliations:** 1 Naval Submarine Medical Research Laboratory, Groton, CT, USA; 2 Department of Health and Kinesiology, Texas A&M University, College Station, TX, USA; 3 Department of Nutrition, Case Western Reserve University School of Medicine, Cleveland, OH, USA

## Abstract

Skeletal muscle protein synthesis has generally been determined by the precursor:product labeling approach using labeled amino acids (e.g., [^13^C]leucine or [^13^C]-, [^15^N]-, or [^2^H]phenylalanine) as the tracers. Although reliable for determining rates of protein synthesis, this methodological approach requires experiments to be conducted in a controlled environment, and as a result, has limited our understanding of muscle protein renewal under free-living conditions over extended periods of time (i.e., integrative/cumulative assessments). An alternative tracer, ^2^H_2_O, has been successfully used to measure rates of muscle protein synthesis in mice, rats, fish and humans. Moreover, perturbations such as feeding and exercise have been included in these measurements without exclusion of common environmental and biological factors. In this review, we discuss the principle behind using ^2^H_2_O to measure muscle protein synthesis and highlight recent investigations that have examined the effects of feeding and exercise. The framework provided in this review should assist muscle biologists in designing experiments that advance our understanding of conditions in which anabolism is altered (e.g., exercise, feeding, growth, debilitating and metabolic pathologies).

## Introduction

Skeletal muscle comprises ~40-45% of the total mass and ~60% of the total body protein in humans [1]; thus, it is no surprise that skeletal muscle accounts for ~30-45% (dependent upon feeding and/or activity) of whole body protein metabolism [2]. As a result of the high metabolic activity of skeletal muscle, research has centered on determining its role in the pathogenesis of many debilitating and chronic diseases (e.g., AIDS, cancer, sarcopenia, starvation, trauma and sepsis and several metabolic disorders). This is accomplished by examining changes that occur in gene expression, transcription factors and signal transduction pathways; however, the structure and function of skeletal muscle may largely be dependent on the renewal of protein. Thus, direct measurement of muscle protein synthesis can provide relevant information regarding the metabolic state of the tissue, and therefore, should be included as an outcome variable when examining anabolic/catabolic conditions.

Direct incorporation methods using tracers such as [^13^C]leucine or [^13^C]-, [^15^N]-, or [^2^H]phenylalnine have traditionally been used to measure tissue specific protein synthesis and are generally accepted for providing reliable measurements. The principal is that by providing a known amount of labeled (tracer) and unlabeled amino acid (tracee) the tracer and tracee will mix with the endogenous pool and become incorporated into protein over time. The tracer and tracee are generally administered as a primed-constant infusion (a priming bolus of tracer and tracee is administered and is continuously provided at a lower concentration to maintain enrichment), or as a flooding dose (a supraphysiological bolus of tracer and tracee is provided over seconds to minutes). Protein synthesis can be determined by measuring the enrichment (tracer/tracee) of the protein against the enrichment of the precursor (i.e., precursor:product labeling ratios). However, two central concerns that have existed with the precursor-product method have been identification and measurement of the true precursor (aminoacyl-tRNA), and the inability to conduct the tracer experiments in a free-living environment over an extended period of time, an integrative/cumulative assessment.

To circumvent the first concern in regards to isolating and measuring the aminoacyl-tRNA (a small pool with a rapid turnover), investigators have used surrogates (i.e., plasma and/or protein-free compartments), or implemented the flooding dose approach. Researchers that have directly compared the labeling ratios of the surrogates to the tRNA-bound amino acid (e.g., leucyl or phenylalanyl) have reported inconsistent findings as to which surrogate most accurately reflects the aminoacyl-tRNA [3-6]. In addition, although the flooding dose [7] can minimize labeling gradients by saturating the uptake of the amino acid by a tissue(s), questions such as whether muscle protein synthesis is stimulated when flooding with specific amino acid tracers [8,9] exists when conducting the experiment in the fasted state.  Therefore, researchers should consider the potential consequences when using various tracers with different approaches (primed-constant infusion *vs*. flooding dose) and interpret the data accordingly (i.e., qualitative comparisons).

The second concern, a controlled setting and short duration tracer infusion protocols, have not allowed for tracer experiments to be conducted under free-living conditions over extended periods of time. More specifically, the experiments require a controlled laboratory environment for the placement of cannulas to infuse the tracers and extract blood, and to insure that steady state enrichments are maintained over the course of the study. In addition, tracer studies are normally conducted following an over-night fast, and if nutrition is provided during the study additional tracer is needed to minimize dilution of the precursor. Although controlled environments are necessary to determine if a specific independent variable(s) influences muscle protein synthesis, one must consider the likelihood that the data will reflect a subject's unnatural behavior.

An alternative, but less familiar approach that can bypass these potential limitations is the use of deuterium oxide (^2^H_2_O), which was first described by Hans Ussing in 1941 [10]. Ussing discovered that by providing ^2^H_2_O to mice and rats, one could determine a rate of protein renewal by the incorporation of ^2^H atoms (i.e., as ^2^H-labeled amino acids) into protein; including liver, kidney, skeletal muscle, and specific proteins such as myosin. He concluded that if ^2^H_2_O was provided to an animal and the concentration was maintained then the ^2^H would accumulate into protein over time allowing for the determination of protein synthesis rates. Ussing thought that this occurred by the transfer of ^2^H with the H of amino acids through transamination reactions and similar processes, which would then be incorporated into protein [10]. Additionally, he suggested that the measurement of this process would be easiest if the exchange between ^2^H with amino acids occurred very rapidly so that their ^2^H content would not change throughout the incorporation process as long as a consistent ^2^H_2_O concentration was maintained [10]. Interestingly, follow-up studies were not published, perhaps due to the difficulty in obtaining deuterium oxide during this time period.

In this review, we will discuss the application of ^2^H_2_O to measure skeletal muscle protein synthesis with known perturbations of interest to the field of muscle biology, feeding and exercise. Emphasis will be placed on the methods to aid researcher's in future experimental design. Although the focus of this review is skeletal muscle protein, the information contained within can be extended to study specific proteins within muscle (e.g., cytosolic, membrane, mitochondria and myofibrillar) or other tissues of interest. Furthermore, the application of ^2^H_2_O can be extended to investigate lipid [11] and glucose kinetics [12], cell proliferation [13], and total energy expenditure (when combined with ^18^O) [14]. Finally, it is not our intention to undermine the numerous investigations that have, and currently employ protocols using labeled amino acids as tracers, as it is our opinion that the experimental question should determine what tracer method to use.

### Mechanism of action

The use of ^2^H_2_O is similar to the use of other precursor:product labeling schemes, a labeled tracer is provided and its incorporation in the product is determined. In the case of ^2^H_2_O, the administered precursor (water) labeling is M+2 yet one measures M+1 in the product. Namely, the di-deutero labeled water that is given rapidly dissociates to yield mono-isotopically labeled water (^2^HHO), thus the equilibration of ^2^H-label with various amino acids (e.g., alanine) leads to the generation of singly-labeled species.

^2^H_2_O has been administered to small animals (mice and rats) by an intraperitoneal injection (~10-20 μl·g body mass^-1 ^with ~99.9% ^2^H_2_O) [15-17], orally to humans (~5 ml·kg body mass^-1 ^with 70% ^2^H_2_O) [18], and to fish simply by addition to the aquarium water (~2.0 to 4.0% ^2^H_2_O) [19]. The goal is to rapidly obtain and then maintain a certain level of ^2^H-labeling in body water (e.g., ~2.0 mole percent excess, MPE, or greater in rodents and fish, and ~0.5% in humans), which allows a steady-state incorporation into end-products. Higher enrichments can be achieved in humans (e.g., ~2.0%), however, a slower increase in ^2^H-labeled body water (no more than 0.5% increase per day) should be considered to minimize the chances of experiencing nausea and vertigo [20,21]. If the purpose of the experiment is to measure synthesis rates over days to weeks, then providing maintenance ^2^H_2_O is recommended and can easily be accomplished by adding the isotope to drinking water [22]. Although ^2^H-labeling can be measured by a variety of methods (e.g., isotope ratio mass spectrometry [13] and nuclear magnetic resonance [23]), we have mainly focused on gas chromatography-mass spectrometry (GC-MS) methods herein since our lab is specialized in those techniques (discussed in "Measurement").

**Figure 1:**
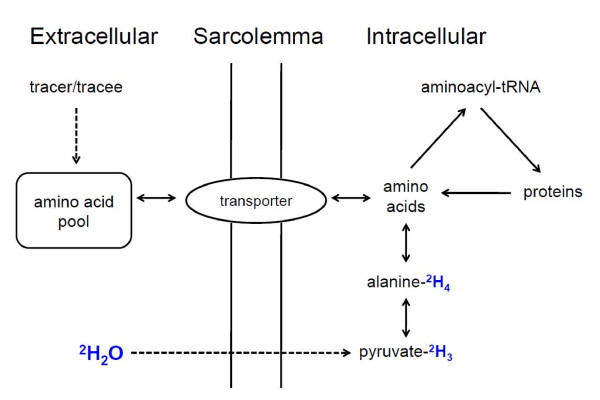
**Fundamental principal of the precursor:product labeling method to determine muscle protein synthesis**. Traditionally, a labeled (tracer) and unlabeled (tracee) amino acid are provided either as a primed-continuous infusion or flooding dose, and mix with the protein-free amino acid pool in the blood. If the fate of the amino acid is incorporation into muscle protein, then entry into the cell is facilitated by amino acid transporters prior to charging an aminoacyl-tRNA. When using ^2^H_2_O, the ^2^H rapidly equilibrates with body water and labels amino acids (e.g., alanine) intracellularly via transamination reactions (e.g., alanine aminotransferase), which can then charge aminoacyl-tRNA and become incorporated into growing peptides.

The uniqueness of using ^2^H_2_O is that, unlike labeled amino acids which must gain entry into the cell via transporters, ^2^H-labeling of carbon-bound hydrogens of amino acids (e.g., alanine) occurs intracellularly (Figure 1) [16,18]. Although all amino acids can become ^2^H-labeled at their α-carbon positions [24], alanine undergoes rapid turnover [25] and possesses 4 potential sites (α - and β-hydrogens) for ^2^H-labeling to occur via transamination [26], thus allowing for easier detection. More specifically, the ^2^H-labeling of the α-hydrogen is approximately equal to that of ^2^H-labeled water and the labeling of total hydrogen is ~3.7 times that of ^2^H-labled water [12,14]. Therefore, it is not the concentration of alanine that determines the percent of ^2^H-lableled alanine, but the percent of the ^2^H-labeled body water. In addition, the carbon-bound hydrogens on alanine do not undergo nonspecific exchange post-translationally or during protein hydrolysis. Thus, labeling gradients are minimized due to rapid equilibration (near 100%) between ^2^H-labeling of body water and alanine [16,18]. As a result of this, precursor (^2^H-labeling of body water):product (either the ^2^H-labeling of the α-hydrogen or total hydrogens of protein-bound alanine divided by "n", the number of incorporated deuteriums) labeling ratios can be determined with certainty. Although we have determined the number of incorporated deuterium's to be ~3.7 in mice [15], rats [16] and humans [18], there was ~0.1 MPE (~3.8) difference in fish [27] suggesting that there may be species- or condition- specific effects on the exchange constant. Thus, there may be differences between species and recommend direct measurement of "n" when examining the rate of protein renewal in other animals.

Another important point that needs to be addressed is the lack of influence that feeding has on ^2^H-labeling gradients. For instance, Dufner et al. [16] provided a supraphysiological bolus of unlabeled alanine (2 g·kg body mass^-1^) to rats following 90 min of ^2^H_2_O administration and measured the ^2^H-labeling of body water, and the α-hydrogen and total hydrogen's of alanine. Although the bolus of unlabeled material affected the plasma concentration of alanine (i.e., it increased from basal values of ~1.2 mM to ~5.5 mM between 120 and 135 min and remained elevated for the duration of the experiment (~2.5 mM at 240 min) the labeling of the α - and the total hydrogens equilibrated in a similar time course. In further support of this, Belleto et al. [28] reported that feeding did not modify the kinetics of plasma protein-free, or plasma protein-bound ^2^H-labeled alanine in both the portal and peripheral circulation. These data suggest that feeding does not result in dilution of the precursor and that the ^2^H-labeling of body water provides an accurate representation of ^2^H-labeled protein-free alanine and is more easily determined. Additionally, there is no reason to believe that a stressor such as exercise would influence ^2^H-labeling gradients and, in fact, we reported similar ^2^H-labeling of body water between exercised and sedentary cage control animals [17]. Even in a disease state, such as chronic kidney failure where fluid changes occur, we did not observe any differences between the ^2^H-labeling of the α-hydrogen of alanine and that of body water [18]. Taken together, experiments can be designed to address questions aimed at determining the influence of perturbations (e.g., feeding and exercise) without concerns of precursor fluctuations.

There may be scenarios where the use of ^2^H_2_O may not be the most appropriate tracer. One specifically is when the hypothesis is aimed at determining the influence of an independent variable(s) on the rate of protein synthesis over a very short period of time (e.g., minutes). In this case, the flooding dose is likely more suitable. More specifically, although ^2^H_2_O rapidly equilibrates with body water and labels alanine, within ~20 minutes [16], time is needed for ^2^H-alalnine to incorporate into protein, which is dependent upon the turnover rate.

### Measurement

**Figure 2:**
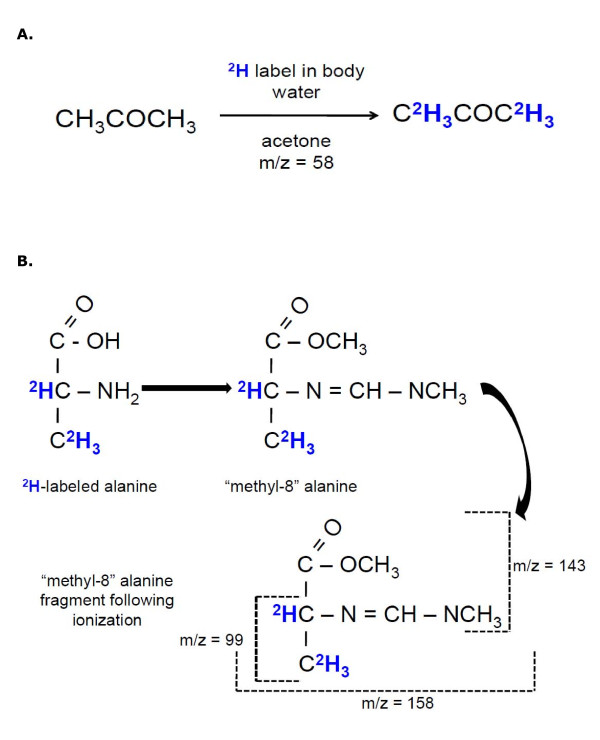
**Measurement of ^2^H-labeling of body water and protein-bound alanine using gas chromatography-mass spectrometry**. Panel A shows that ^2^H-labeling of body water (plasma) is determined by ^2^H exchange with carbon-bound hydrogens of acetone. Acetone has a mass-to-charge ratio (*m/z*) of 58, thus *m/z *59 represents ^2^H-labeling. Panel B shows the methyl-8 derivative upon reacting the protein hydrolysate with *N, N-*dimethylformamide dimethyl acetal. Three ions that have been characterized are *m/z *99 (the base peak that includes α and β-hydrogens of alanine), *m/z *143 (includes only the α-hydrogen of alanine) and *m/z *158 (represents the molecular ion). Since the precursor (water) labeling is relatively low (e.g., 0.5 to 2%) one typically only measures changes in M+1/M0 ratio, although in theory M+2, M+3, etc. mass isotopomers are generated, their abundance is typically not measurable (e.g., when the water labeling is 0.5%, the abundance of M+6 acetone is ~0.005^6^, likewise the abundance of M+4 alanine is ~0.005^3.7^).

A detailed description of sample preparation and GC-MS programming is beyond the scope of this review, thus we refer readers to the following papers [16-18]. In short, a minimum of 10 to 20 μl of plasma and ~5 mg of mixed muscle are needed for measurement of the ^2^H-labeled/unlabeled ratios by GC-MS. There are, however, times when more muscle tissue is needed (30-100 mg), such as when specific fractions or proteins within muscle (e.g., mitochondria, myofibrillar, sarcoplasmic, actin, myosin, etc.) are being examined. For quantification, we analyze the ^2^H enrichment of acetone following exchange with the ^2^H-labeling of body water, Figure 2A[29] and the "methyl-8" derivative of the protein bound ^2^H-labeled alanine after reacting the hydrolysate with *N, N*-dimethylformamide dimethyl acetal, Figure 2B[30]. The GC-MS is operated in electron impact ionization mode (70 eV) and selective ion monitoring of mass-to-charge ratios (*m/z*) of 58 (*M*) and 59 (*M *+ 1) for body water, and 99 (*M*) and 100 (*M *+ 1) for protein-bound alanine using a dwell time of 10 ms per ion is conducted. Thus, the *m/z *ratios 59/58 and 100/99 represent the precursor and product, respectively, and are expressed as "% labeling". As a result of there being naturally occurring isotopes within organisms (i.e., ^2^H is ~0.015%, ^13^C is ~1.1% and ^15^N is ~0.4%), the fragment ion ratio *m/z *100/99 (of the derivative) yields ~6.5% natural background labeling. As implied herein, the use of stable isotopes do not require parallel measurements of the tracer (alanine) concentration since the GC-MS simultaneously measures the labeled and unlabeled molecules, allowing for immediate calculation of the ratio, dissimilar to radioisotopes which require knowledge of the total concentration of the amino acid for determination of the specific activity.

An important question that investigators may consider a concern, "What affects the limit of ^2^H_2_O that can/should be administered?" requires a consideration of physiological and analytical factors. First, although we have typically maintained less than 3% ^2^H in body water in rodents, it appears that one can maintain ~5% ^2^H-labeling in body water for prolonged periods in rodents without any negative influences/side effects. In humans, nausea and vertigo have been reported in subjects who ingested a dose of ^2^H_2_O sufficient to acutely enrich body water to ~0.5%. These transient side effects can be avoided by instructing subject to ingest a few smaller doses over a short time period; for example, if the goal is to have a subject drink 200 ml of ^2^H_2_O, rather than give this is a single glass of water, subjects may drink 50 ml every 30 min. In regards to concerns for analytical windows, providing more tracer will obviously yield more ^2^H-labeling in products over a given time. At the dose we have been using (e.g., ~0.5% in humans and ~2% in rodents) there is reasonable incorporation into mixed muscle proteins. To improve the quantification of low levels of ^2^H-labeling in products (e.g., ~0.15% excess labeling), one can selectively integrate ion chromatograms [12]; an alternative is to use GC-pyrolysis-IRMS [13].

Another concern regarding the use of ^2^H_2_O centers on the half-life of the tracer and the ability to repeat studies. For example, the half-life of ^2^H in body water in healthy humans is ~7 to 10 days. Therefore, one might consider ~2 months before repeating an investigation in the same subject(s) if the goal is to allow ~5 half-lives to elapse and eliminate most of the tracer. Although we are not aware of any cross-over studies regarding protein synthesis in which subjects were studied more than at one time, we have previously demonstrated that one could measure gluconeogenesis by performing 'back-to-back' experiments in rats given ^2^H_2_O [12]. Namely, although the half-life of water in a rat is ~3-4 days, we were able to administer a low dose of ^2^H_2_O on day 1 (e.g., to enrich the pool to ~0.05%) and then a higher dose of ^2^H_2_O on day 2 (e.g., to enrich the pool to ~0.5%). We expect that this logic would be practical to use in studies of protein synthesis, especially if one had access to a GC-pyrolysis-IRMS instrument [13].

### Calculations

**Figure 3:**
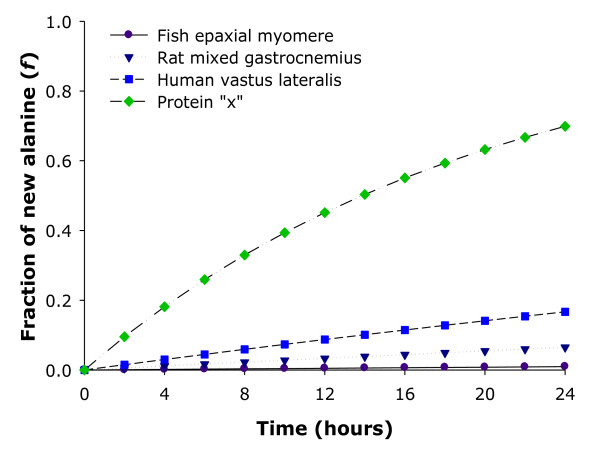
**Theoretical prediction model based on fractional synthetic rate (FSR) measurements made over 24 h**. The fraction of new alanine (*f*) synthesized in mixed muscle of fish (epaxial myomere), rats (mixed gastrocnemius) and humans (vastus lateralis), and a hypothetical protein that has a rapid renewal rate (protein "x"). As a result of skeletal muscle having a slow renewal rate, calculating a FSR with the traditional precursor:product labeling equation provides a reliable estimate (i.e., there is no significant difference in the values when using rise-to-plateau kinetics, F_t_). However, when proteins have a rapid renewal rate (e.g., protein "x"), the affect of long sampling intervals begins to have substantial effects on estimates of FSR. Thus, it is recommended that the labeling protocol be either reduced or multiple samples be collected with calculations made using *F*_*t *_= f_max _× (1-e^-kt^).

The measurement is the rate at which ^2^H-labeled alanine is incorporated into muscle protein(s) relative to the total abundance of the alanine pool per unit of time (fractional synthesis rate, FSR) and can generally be calculated by the equation:(1)

where, *MPE *(mole percent excess)_*Ala *_represents the total ^2^H-labeling of protein-bound alanine, "*n*" represents the exchange of ^2^H between body water and free alanine (~3.7 in mammals and ~3.8 in fish), *MPE*_*BW *_represents the labeling of body water and *t *is time [16]. The assumptions are that the ^2^H-labeled body water equilibrates with free alanine more rapidly than ^2^H-labeled alanine becomes incorporated into newly made protein and that synthesis is constant over the course of the experiment (i.e., any changes in synthesis are associated with a change in ^2^H-label incorporation) [16,31]. There are, however, two scenarios that would require synthesis rates to be calculated by estimating fractional protein synthetic rate constants by non-linear least squares fitting of plots (rise-to-plateau kinetics):(2)

where *f*_*max *_is the fraction, or percent, of new ^2^H-labeled alanine incorporated into protein, *k *is the predicted fractional synthesis rate constant and *t *is labeling time. One scenario is when the assessment is made over an extended period of time (several days to months) and another is if a specific protein with a known high turnover rate is being examined (i.e., skeletal muscle is heterogeneous pool). Figure 3 is a theoretical prediction model based on measurements that were obtained using equation 1 after 24 h of ^2^H_2_O exposure in mixed muscle of fish [19], rats [17] and humans (unpublished observations), and a hypothetical protein that has a rapid renewal rate (protein "x"). In each of our experiments, the expected FSR in fish, rats and humans (0.04, 0.28 and 0.78%·h^-1 ^respectively) closely resembled the F_t_'s (0.04%, 0.27% and 0.70%·h^-1 ^respectively). Therefore, the values are still in the early, and linear, part of the curve and are far from the "plateau" portion, thus reasonable estimates of FSR are obtained. In contrast, if the protein has a high renewal rate (i.e., protein "x" near 5.0%·h^-1^), the affect of long sampling intervals begins to have substantial effects on estimates of FSR (using a single time point later in the experiment). Taken together, if the aim is to measure mixed muscle or the myofibrillar fraction, which comprises 70% of the total protein pool [32], use of the traditional labeling equation (equation 1) is reasonable for calculating FSR. However, when one is interested in proteins that have rapid renewal rates within skeletal muscle (e.g., specific cytosolic proteins), or if the course of the experiment is over days to weeks, then several samples should be obtained and the FSR calculated by using rise-to-plateau kinetics (equation 2).

### Effects of feeding & exercise

As previously mentioned, examining the effects of feeding and exercise on muscle protein synthesis can often present challenges to the investigators. Using ^2^H_2_O offers advantages in that experiments can be designed to either directly measure the effects of these indirect variables on FSR, or include the normal biological and environmental factors that routinely occur in organisms on the measurements over time, an integrative/cumulative assessment. Although it is common knowledge that nutrients and load bearing exercise stimulate muscle protein synthesis, it is not known whether the effect persists, and to what degree when measured under free-living conditions. As a result of this, our group has focused on examining the effects of feeding and exercise (encompassing normal daily routines) to determine their "true" impact on muscle FSR in different organisms (Table 1).

**Table 1:** Using ^2^H_2_O to study the effects of feeding and exercise on muscle protein synthesis

Objective	Species	Assessment time	Outcome	Reference
Determine if obesity impairs protein synthesis following nutrient ingestion	C57BL/6J mice	5 hours	Diet-induced obesity impairs nutrient stimulated MPS	[26]

Determine the effects of acute fasting & chronic food restriction on protein synthesis	Male Wistar rats (2-3 mo old)	6 hours	Acute fasting (20 h) and chronic food restriction (7 d) impaired MPS	[27]

Compare flooding dose vs. ^2^H_2_O to measure protein synthesis with acute resistance exercise	Male Sprague- Dawley rats (5-6 mo old)	4 & 24 hours	Methodologies compare qualitatively, and more muscle protein is synthesized over 24 h vs. 4 h, however, synthesis rates were higher when measured over 4 hours	[20]

Determine if chronic resistance exercise attenuates acute exercise stimulated protein synthesis	Male Sprague- Dawley rats (5-6 mo old)	36 hours	Resistance exercise training alters the anabolic (MPS) response to acute exercise	[28]

Determine the effects of high-intensity resistance exercise when energy & macronutrient content controlled	Male Humans (20-24 y old)	24 hours	High-intensity resistance exercise stimulated myofibrillar, but not mixed MPS	[†]

† Unpublished Observations. Muscle protein synthesis, MPS.

To date, there have only been two investigations that have measured the acute effects of feeding on muscle protein synthesis using ^2^H_2_O [33,34]. In the first experiment [33], the purpose was to determine whether diet- induced obesity impairs tissue-specific FSR (a somewhat controversial topic) in C57BL/6 mice following 9-weeks of consuming either a high-carbohydrate or high-fat diet. In the second experiment [34], the rationale was to examine the effects of acute fasting (20 h) *vs*. chronic food restriction (7 days) on FSR in rats. In both designs, the FSR measurements were made over 5-6 h with feeding, since there is minimal concern for labeling gradients when using ^2^H_2_O, data were obtained with a certain degree of confidence. Both studies provided new insight into two feeding extremes: *1) *diet induced obesity results in an attenuation in the feeding induced stimulation of muscle protein synthesis in mice, and *2) *both acute fasting and chronic food restriction significantly reduce muscle protein synthesis in rats.

In regards to examining the effects of exercise (resistance based) with ^2^H_2_O, our group has conducted several experiments in rats [17,27] and, most recent, in humans (unpublished observations). The questions that our group wanted to address using ^2^H_2_O that we could not with other isotopes alone were: *1*) is a single bout of high-intensity resistance exercise anabolic in rats, *2) *does chronic resistance exercise result in an anabolic "plateau", and *3) *does a single session of high-intensity resistance exercise in humans stimulate muscle protein synthesis when energy and macronutrient ingestion is controlled and the experiment is conducted in the fed state. In the first experiment [17], we measured FSR over 25 min with L-[2,3,4,5,6-^3^H] phenylalanine (flooding dose) and with ^2^H_2_O over 4 and 24 h with an exhaustive session of resistance exercise. Though the conclusions drawn were not different with any of the measurements made (i.e., there was no exercise effect), we determined that measurements made over various time points reveal different values. More specifically, the FSR from the 24 h measurements were less than the 4 h measurements when expressed as %·h^-1^; however, a greater percentage of the pool was obviously synthesized over 24 h. As a result of these data, results from different tracer protocols should be compared "qualitatively," which was suggested by Smith et al. [35] following their observations of a 20% increase in FSR with [5,5,5-^2^H_3_]leucine *vs*. [^2^H_5_]- and [^13^C_6_]phenylalanine. Additionally, the expression of FSR over the course of a tracer experiment (e.g., % renewed over 24 h *vs*. %·h^-1 ^when measured over 24 h) may better explain the metabolic state of the tissue. In our second experiment [27], we measured muscle protein synthesis at a time that has been determined to be anabolic in rats following resistance exercise (16 h following the previous bout [36]) using L-[2,3,4,5,6-^3^H] phenylalanine and observed no differences from the control groups; however, we observed significant elevations in FSR with ^2^H_2_O measured over 36 h. Thus, had we not employed an integrative assessment using ^2^H_2_O we would have falsely concluded that chronic resistance exercise training diminishes the anabolic effect. In our third study (unpublished observations) we assessed the effect of a high-intensity bout of resistance exercise in the fed state with energy and macronutrients controlled over 24 h in humans. Although we did not find a stimulatory effect of exercise on FSR in the mixed vastus lateralis, there was a significant effect on the myofibrillar FSR with exercise. Thus, the findings from this study emphasize that skeletal muscle protein is a heterogeneous pool, and although mixed muscle should be reflective of the myofibrillar fraction, this may not always be the case; we expect that future studies should focus on specific fractions or proteins within skeletal muscle.

## Conclusions

The use of ^2^H_2_O is perhaps the oldest [10] and least invasive method for determining muscle protein synthesis, yet has not been regularly employed. There is considerable interest as to the rate at which muscle protein(s) are renewed over extended periods of time with inclusion of normal biological processes and environmental influences, and these experiments can be pursued with ^2^H_2_O. Our group's experiments have provided a starting point for determining the effects of feeding and exercise on "integrative" muscle protein synthesis, and hope that our description of the fundamental principles behind using ^2^H_2_O will assist others with its application to address questions of interest to the field.

## Competing interests

The authors declare that they have no competing interests.

## Authors' contributions

All authors contributed equally to this article, read and approved the final version of the manuscript.
